# The Influence of Parents’ Educational Expectations on Children’s Development: The Chain Mediation Role of Educational Anxiety and Parental Involvement

**DOI:** 10.3390/bs14090779

**Published:** 2024-09-05

**Authors:** Ye Xin, Lu Yu

**Affiliations:** 1 Faculty of Education, The University of Hong Kong, Central and Western District, Hong Kong SAR 999077, China; xinye2001@connect.hku.hk; 2 Faculty of Education, Southwest University, Chongqing 400715, China

**Keywords:** educational expectations, educational anxiety, child development, parental involvement, educational involution

## Abstract

In the social context of the “Educational Involution”, the educational expectations of parents have a potential influence on the development of children. High parental educational expectations create parental anxiety, which in turn results in a rise in parental involvement and eventually promotes the growth and progress of children. The current study administered an electronic questionnaire to 891 parents of young children in four provinces of China. The questionnaire included the Parental Educational Expectations Scale, the Educational Anxiety Scale, the Parental Involvement Scale, and the Child Development Scale. This study used SPSS 27.0 for statistical data analysis and the SPSS macro program PROCESS to explore the mediation role. We found that (1) educational anxiety plays a mediating role between parental educational expectations and child development; (2) parental involvement has a mediating effect between parental educational expectations and child development; and (3) educational anxiety and parental involvement play a chain mediating role between parental educational expectations and child development. In conclusion, parental educational expectations appear to contribute to child development, and this effect may be mediated individually and sequentially by educational anxiety and parental involvement.

## 1. Introduction

In recent years, China’s economic development has decelerated, leading to a shortage of quality educational resources. This has created a contradiction, where the growth rate of educational demand exceeds the growth rate of available educational resources [1], resulting in the intensification of the social phenomenon of “educational involution”. In addition, our country’s cultural context fosters “high educational expectations”, which include the concept of “feeding-back” educational expectations (children are expected to repay their parents when they grow up) [2]. Moreover, cultural traditions such as the saying “a good scholar can become an official” emphasize the importance of education for securing a good job [1]. Similarly, the expression “anticipating one’s son becoming a dragon and one’s daughter becoming a phoenix” reflects the universal desire of parents for their children to succeed. All these factors collectively contribute to the formation of high expectations about the education of parents [3]. Notably, parents’ elevated educational aspirations are not limited to primary and secondary education, but also extend to the kindergarten stage [4,5,6]. Hence, it is imperative to give careful consideration to the educational expectations of parents with young children.

Expectation theory suggests that expectations from significant others can be effective in promoting adolescents’ intelligence, academic achievement, and social behavior. One well-known phenomenon in this context is the “Rosenthal Expectancy Effect” [7]. When parents have elevated educational aspirations for their children, it often translates into increased recognition and encouragement for their offspring [8]. Notably, studies have demonstrated that maintaining educational expectations within a reasonable threshold can yield positive effects, akin to the “Rosenthal Effect”. This effect contributes to children’s overall development and enhances their quality of life [9,10].

At the same time, parental educational expectations exert a multifaceted impact on various aspects of child development. For instance, they correlate with adolescents’ academic performance, learning involvement, and educational achievement [4,11,12,13]. Given these findings, it is essential to conduct further analysis and discussion regarding the intricate correlation between educational expectations of parents and children’s growth. However, an intricate relationship exists between educational aspirations of parents and children’s growth, necessitating exploration through extensive research. The existing literature suggests that parental educational expectations correlate with several factors, including educational involvement, educational anxiety, and children’s cognitive abilities [6,14,15]. Child development is a multifaceted concept encompassing psychological, physical, and social dimensions. It is influenced by various factors, such as parenting styles, educational involvement, and parental co-parenting [16,17,18,19,20]. Additionally, researchers have delved into mediating variables that connect parental educational expectations to child development. These variables include learning pressure, educational involvement, and children’s self-expectations, leading to the construction of mediating effects [21,22]. Nonetheless, there is no research that discusses the underlying mechanisms between parental educational expectations and child development and which treats educational anxiety and parenting involvement as mediating variables. In light of this, our present study aims to develop a multilevel model which, for the first time, explores the potential influence of educational expectations of parents on child development through the mediation role of educational anxiety and parenting involvement. By doing so, we seek to provide a theoretical foundation for promoting optimal child growth and well-being.

### 1.1. Relationship between Educational Expectations of Parents and Child Development

Scholars have approached the definition and conceptualization of parental educational expectations in different ways. On the one hand, a portion of scholars consider parental educational expectations as a single expectation for a certain aspect of the child, such as the final degree of education, future educational achievement and strategies and blueprints for children’s future based on the parents’ own experiences [23,24,25,26]. Most of the studies simplify expectations about the education of parents to parental aspirations of their children’s attainment of education. On the other hand, some scholars believe that parental educational expectation is a comprehensive concept. It consists of five dimensions, namely, academic performance, future development, interpersonal relationships, behavioral performance, and physical and mental qualities [12]. These dimensions encompass expectations related not only to educational achievements but also to skills, occupations, and other facets of a child’s growth.

Importantly, parental educational expectations are directly related to the objectives and orientations of family education, and serve as a driving force for children’s actions [27]. Based on the above research, this study contends that parental expectations extend beyond educational attainment alone. It should be a comprehensive concept that includes parents’ expectations of their children’s behavior, interpersonal relationships, and other aspects. Therefore, this research defines educational expectations of parents as an attitude held by parents of young children toward their offspring’s future development, including parents’ expectations about academic achievement and behavioral expectations, and their future developmental expectations for their children [28].

Child development is a complex and comprehensive concept that includes diverse facets of children’s cognitive, behavioral, and social development. Most scholars tend to focus on one aspect of the growth of children, such as cognition, language, and behavior [29,30,31]. However, this study contends that child development should be analyzed in a comprehensive manner. Therefore, we define child development as all aspects of child development, including learning, sociality, and physical and mental health.

A growing body of researches have explored the correlation between parental educational expectations and child development. Zhou emphasized the fact that educational aspirations of parents can promote the progress of children’s cognitive ability. Specifically, the higher the educational expectations of parents, the stronger the cognitive ability in children [15]. Meanwhile, other scholars also pointed out that educational aspirations of parents can positively impact the development of children’s math achievement. These educational expectations have a strong positive predictive effect on the math achievement of children [32,33]. Based on this, the present research proposes the following hypothesis.

**Hypothesis 1:**  *Educational expectations of parents possibly predict child development.*

### 1.2. Relationship between Educational Anxiety, Educational Expectations and Child Development

Educational anxiety refers to a persistent emotional state, such as anxiety, nervousness, and worry, produced by parents throughout the process of instructing their children. This anxiety stems from the ambiguity surrounding their children’s learning, choosing schools, and employment [34,35]. Educational anxiety mainly includes the dimensions of academic performance anxiety, learning attitude anxiety, and future development anxiety [36]. Research has discovered that there is a correlation between educational aspirations of parents and educational anxiety. Notably, higher parental educational expectations are often accompanied by the generation of higher educational anxiety. As pointed out by Fan and other scholars, in their study, educational aspirations of parents commence at an early stage in a child’s life and progressively intensify during the kindergarten years, leading to parental anxiety [6].

An extensive body of research indicates that parental educational anxiety significantly affects children’s growth. On the one hand, parental educational anxiety positively predicts academic burnout [37]. On the other hand, studies have revealed that although excessive parental educational anxiety negatively affects children’s learning quality, especially in children’s learning in math-related subjects, moderate anxiety can be equally effective in family environments with good parenting styles and higher educational involvement [38]. Thus, educational anxiety may be an important mediating mechanism in explaining the correlation between educational expectations and child development. While this idea has not been directly tested, the existing research evidence indirectly supports the mediation role of educational anxiety in this relationship. In summary, the following hypothesis is proposed for our study.

**Hypothesis 2:**  *Educational anxiety possibly mediates the relationship between educational expectations of parents and child development.*

### 1.3. Relationship between Parental Involvement, Educational Expectations and Child Development

Parental involvement refers to all activities in which parents are involved that directly or indirectly affect children’s development [39]. It includes four dimensions: support and planning, daily care, encouragement and praise, and discipline and restraint [40]. Many scholars have found in their studies that expectations about the education of parents have a certain impact on parental involvement. As a matter of fact, educational aspirations, as the psychological expectations of fathers and mothers for their children’s educational achievement, are the intrinsic motivation for families to invest in education [41]. Simultaneously, educational expectations can shape the mode of a family’s investment in education and the effect of nurturing [3]. The more parents’ expectations increase, the more attention they devote and the greater the allocation of resources towards their children’s education [42].

Parental involvement has an impact on children’s development. In their study, Hou and other scholars pointed out that a father’s parenting involvement significantly predicted children’s emotional adjustment, while a mother’s parenting involvement predicted children’s behavioral adjustment [43]. Additionally, other scholars have indicated that a father’s parenting involvement has an irreplaceable role in promoting children and adolescents’ cognitive development, social development, mental health, personality psychology, and prosocial behavior [44,45,46,47,48]. Furthermore, some studies suggest that family parenting involvement mediates the correlation between the educational aspirations of parents and the academic performance of children who are left behind. Therefore, they advocate the enhancement of academic expectations of children who have been left behind and academic performance by strengthening family parenting involvement [42]. Based on these findings, the following hypothesis is proposed in this research.

**Hypothesis 3:**  *Parental involvement serves as a potential mediator in the connection between parental educational expectations and child development.*

### 1.4. Potential Chain Effects of Educational Anxiety and Parental Involvement

Numerous studies have established a link between educational anxiety and parental involvement. When parents experience high educational anxiety, they tend to alleviate this anxiety by increasing parenting involvement. As scholars such as Gong indicated in their research, family educational anxiety positively influences extracurricular tutoring input [49]. Scholars at home and abroad have pointed out that as educational expectations increase and the level of parental educational anxiety deepens, families will correspondingly increase the cost of child rearing, ultimately promoting children’s development [50,51].

Currently, several scholars have provided preliminary evidence of the correlation between educational expectations of parents, parental involvement, and child development [42]. And these findings indicate that educational anxiety positively predicts parental involvement, suggesting that child development can be promoted by increasing parenting involvement [49]. However, no studies have directly tested the hypothesis that educational anxiety and parental involvement mediate the correlation between expectations about education of parents for children and children’s development. Advances in this study will help deepen the academic understanding of the impact of educational aspirations of parents on the development of children. Based on this, the subsequent hypothesis is proposed for this research.

**Hypothesis 4:**  *Educational anxiety and parental involvement possibly mediate the correlation between educational expectations of parents and child development.*

**Figure 1:**
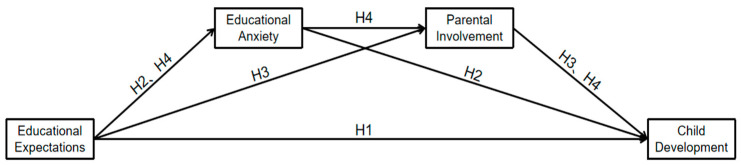
The research hypothesis model.

In accordance with the literature review mentioned above, this research proposes the above hypotheses and constructs a study hypothesis model (Figure 1) of parental educational expectations, educational anxiety, and parental involvement in children’s development. The aim is to explore how educational expectations of parents affect children’s development.

## 2. Materials and Methods

### 2.1. Research Design

This study is an empirical study, and we use the quantitative method for analysis. This study takes parents of young children as the research object and conducts a questionnaire survey with them to understand the real situation of parents’ educational expectations, educational anxiety, parenting involvement and child development, to explore the influence of parents’ educational expectations on child development, and to discuss the mediating role of educational anxiety and parenting involvement. The specific research steps were as follows: first, we reviewed and searched the related literature and wrote a literature review, to lay a theoretical foundation for conducting this study. Second, a questionnaire survey was conducted on the status of parents’ educational expectations, educational anxiety, parenting involvement and child development. Third, the results of the survey were analyzed and discussed.

### 2.2. Participants

For this study, parents of young children in four provinces in China (Sichuan, Chongqing, Liaoning, and Xinjiang) were selected, employing a cluster sampling method. Specifically, the kindergartens were grouped according to their geographical location, and then a certain group of kindergartens was randomly selected, and finally a questionnaire was administered to the parents of this group of kindergartens. Before collecting data, we sought informed consent from each participant, and all parents voluntarily participated in the questionnaire. Parents could stop answering at any time during the process of completing the questionnaire. Additionally, parents were aware that their data would be used for research purposes and that they would not receive any benefit from the study.

A digital survey was distributed to parents in May 2024 via the “Questionnaire Star” platform. A total of 891 parents of young children took part in the questionnaire survey. The criteria for participant inclusion/exclusion were being parents of young children and the ability to understand and complete the electronic questionnaire independently. After excluding questionnaires with potential interferences such as insufficient response time, incomplete information, and identical answers, a total of 767 questionnaires were collected, yielding a sample validity percentage of 86.1%. This research received approval from the Research Ethics Committee of Southwest University and followed the principles outlined in the Declaration of Helsinki.

Of the 767 parents interviewed for this study, 403 (52.5%) were raising boys and 364 (47.5%) were raising girls. The distribution across regions was as follows: 276 (36.0%) in Sichuan Province, 164 (21.4%) in Chongqing Municipality, 156 (20.3%) in Liaoning Province, and 171 (22.3%) in Xinjiang Uygur Autonomous Region. Table 1 presents more comprehensive information of the demographic characteristics of the participants in this research.

### 2.3. Measurement

#### 2.3.1. Measurement of Educational Expectations

The educational expectations scale was originally developed by Li [52]. For this study, certain questions of the scale were adjusted according to the research needs. The scale comprises 29 items that are categorized into five distinct dimensions: learning expectations, sociality expectations, self-care expectations, physical -and mental-quality expectations, and specialty-strength expectations. The dimension scores are the average of the items associated with each dimension. The scale employs a 5-point scoring system, where 1 means “very much not in line” and 5 means “very much in line”. Higher scores indicate higher parental educational expectations in this dimension. The scale exhibits a well-defined structure and demonstrates scale validity, while the internal consistency coefficient is deemed satisfactory. The reliability and validity of the scale were assessed, yielding a Cronbach’s alpha coefficient of 0.952 and a KMO value of 0.958.

#### 2.3.2. Measurement of Educational Anxiety

The educational anxiety scale was originally developed by Chen and other scholars [53]. The scale comprises 12 items distributed across six dimensions: school performance, physical condition, safety condition, psychological condition, future, and teacher condition. The dimension scores are calculated as averages of the items associated with each dimension. The scale employs a 5-point scoring system, where 1 means “very much not in line” and 5 means “very much in line”. Higher scores indicate more severe parental educational anxiety on this dimension. The scale exhibits a well-defined structure and validity, and the internal consistency coefficient is deemed satisfactory. The reliability and validity of the scale were assessed, yielding a Cronbach’s alpha coefficient of 0.939 and a KMO value of 0.916.

#### 2.3.3. Measurement of Parental Involvement

The parental involvement scale was originally developed by Fantuzzo and other scholars, and localized and revised by Chinese scholars Liu and other scholars [54,55]. This study adapted certain questions of the scale according to the research objectives. The scale includes 20 items distributed across three dimensions: home–school communication, participation at school, and participation at home. The dimension scores are the average of the items associated with each dimension. The scale employs a 5-point scoring system, where 1 means “very much not in line” and 5 means “very much in line”. Higher scores indicate more parental involvement in this dimension. The scale has sound structure and validity, and the internal consistency coefficient is satisfactory. The reliability and validity of the scale were assessed, yielding a Cronbach’s alpha coefficient of 0.946 and a KMO value of 0.935.

#### 2.3.4. Measurement of Child Development

The child development scale was originally developed by Li [52]. For this study, certain questions within the scale were adjusted to align with the research objectives. The scale comprises 29 items that are categorized into five dimensions: learning, social interaction, self-care, physical and mental well-being, and specialty skills. The dimension scores are calculated as the average of the items associated with each dimension. The scale employs a 5-point scoring system, where 1 means “very much not in line” and 5 means “very much in line”. Higher scores indicate better development of the child in this dimension. The scale exhibits a well-defined structure and demonstrates scale validity, while the internal consistency coefficient is deemed satisfactory. The reliability and validity of the scale were assessed, yielding a Cronbach’s alpha coefficient of 0.970 and a KMO value of 0.968.

### 2.4. Statistical Analysis

This research employed SPSS 27.0 for the purposes of data input, arrangement, and analysis. Our analytical methods included descriptive statistical analysis, one-way ANOVA, independent samples *t*-test, correlation analysis, and regression analysis based on the questionnaire data. In addition, the SPSS macro program PROCESS (Model 6) was applied to investigate the mediating role of educational anxiety and parental involvement in the link between parental educational expectations and child development [56].

## 3. Results

### 3.1. Descriptive Statistics, Correlation Analysis and Test Analysis Results

#### 3.1.1. Common Method Variance

Prior to conducting data analysis, we conducted a test to detect any potential common method bias. Harman’s one-factor method produced 14 factors that had eigenvalues greater than 1. The initial component explained 30.58% of the variance, which is below the critical threshold of 40%. Hence, this study does not present any substantial indication of methodological bias.

#### 3.1.2. Descriptive Statistics and Correlation Analysis

Table 2 provides the results of the descriptive statistical analyses, encompassing means, standard deviations, and relationship between the variables. Pearson’s correlation coefficient was used to measure the correlation between parental educational expectations, educational anxiety, parental involvement and child development.

As shown, there is a strong positive link between educational expectations and child development (r = 0.386, *p* < 0.001). There exists a strong positive link between educational expectations and educational anxiety (r = 0.196, *p* < 0.001) and educational expectations and parental involvement (r = 0.407, *p* < 0.001). Additionally, there is a strong positive link between child development and educational anxiety (r = 0.311, *p* < 0.001) and child development and parental involvement (r = 0.635, *p* < 0.001). There is a strong positive link between educational anxiety and parental involvement (r = 0.281, *p* < 0.001).

Lastly, it is important to note that varying degrees of correlations exist between family socioeconomic status (SES) and the study variables (educational expectations, educational anxiety, parental involvement and child development). Consequently, the effect of family socioeconomic status (SES) on the study variables needs to be excluded in subsequent studies.

#### 3.1.3. One-Way ANOVA and Independent Samples *t*-Tests

Demographic variables among parents and children exhibited specific differences related to factors such as educational expectations and child development. To avoid the effect on key variables, we used one-way ANOVA and independent samples *t*-tests to examine the presence of intergroup disparities in demographic characteristics on the study variables. The two variables were tested for normal distribution: the skewness of parents’ educational expectations was −1.79 and the kurtosis was 7.82; the skewness of children’s development was −0.57 and the kurtosis was −0.09. The absolute value of the skewness of the two variables was less than 3 and the absolute value of the kurtosis was less than 10, which was basically in line with the normal distribution, and it was possible to carry out the *t*-test and the analysis of variance (ANOVA). According to the spherical test, the values of the spherical test between parental educational expectations, child development, region and class level are less than 0.01. After the test of homogeneity of variance, it was found that the significance of the mean of region and class level was more than 0.05. Therefore, it is suitable for analysis of variance. Multiple comparisons were conducted using the least significant difference (LSD) method. Results showed statistically significant variations in parental expectations for their children’s education by child’s class level. Additionally, we observed statistically significant differences in child development related to child gender and region (Table 3).

### 3.2. Mediation Analysis Results

#### 3.2.1. Regression Analysis

Previous studies and the results of the above tests have indicated that variables such as child gender, region, and family socioeconomic status (SES) are important factors affecting child development. Therefore, child gender, region, and family socioeconomic status (SES) must be considered as control variables. Specifically, we transformed child gender and region into dummy variables and included them in the regression model for analysis. Before performing the regression analysis, we tested the variables for multicollinearity, and the VIF values were all much less than 10; therefore, there was no multicollinearity problem. Through the homoskedasticity test, we have found out that there is no clear trend or pattern in the residual plot. Therefore, the assumption of homoskedasticity is satisfied.

The regression analysis revealed that parental expectations for education had a strong positive effect on the development of children (β = 0.386, *p* < 0.001) (Table 4). Put simply, when parents have higher educational expectations for their children, it leads to improved child development. Moreover, the study’s findings signified that child gender, region, and family socioeconomic status (SES) showed statistical significance as control variables. This finding indicates that child gender, region, and family socioeconomic status (SES) are important variables to be considered in understanding the influence of educational expectations on the development of children. 

**Figure 2:**
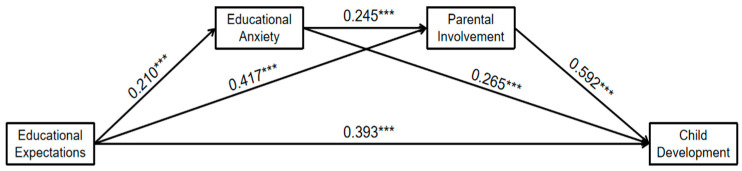
The mediation model. Note: *** *p* < 0.001.

A chain mediation model was established, with educational expectations as the independent variable, child development as the dependent variable, and educational anxiety and parental involvement as the mediating variables (Figure 2). It was discovered that a considerable direct effect existed between educational expectations, educational anxiety, parental involvement and child development. 

Specifically, parents’ educational expectations positively predicted educational anxiety (β = 0.210, *p* < 0.001), parental involvement (β = 0.417, *p* < 0.001), and child development (β = 0.393, *p* < 0.001). Furthermore, educational anxiety accurately predicted parental involvement (β = 0.245, *p* < 0.001) and child development (β = 0.265, *p* < 0.001). Lastly, parental involvement accurately predicted child development (β = 0.592, *p* < 0.001) (Table 5).

Thus, Hypothesis 1 of this study has been validated. In addition, we observed that gender, region, and family socioeconomic status (SES) impact parents’ expectations for education, educational anxiety, parental involvement, and child development.

#### 3.2.2. Analysis of Mediation Effects

This research investigated the correlation between educational expectations (independent variable) and child development (dependent variable) with two mediation variables: educational anxiety and parental involvement. The selection of Model 6 in the PROCESS macro program was based on the method of confidence interval. The findings indicated that the total impact value was 0.625, the direct effect value was 0.237, and the total mediation effect value was 0.387 (Table 6). 

The confidence interval test assessed the correlation between child development, educational expectations, educational anxiety, and parental involvement. The findings indicated that the mediating effects of “educational expectations–educational anxiety–child development” and “educational expectations–parental involvement–child development” had 95% confidence intervals, which exclude zero. This implies that educational anxiety and parental involvement play a mediation role in this relationship. 

In the mediation analysis, the coefficient of the educational anxiety variable is 0.041, representing a contribution of 6.7% to the overall effect. Moreover, the parental involvement variable has a coefficient of 0.314, which accounts for 50.2% of the overall effect. The significant importance of parental involvement in mediating is apparent, and accounts for a substantial proportion. Pathway analyses suggest that young children’s parents with higher educational expectations develop greater educational anxiety, which in turn contributes to child development. Simultaneously, parents who have greater expectations for their children’s education tend to increase their investment in all aspects of parenting, to enhance their children’s development. 

Therefore, Hypotheses 2 and 3 of this study have been validated. The analysis indicates that the 95% confidence interval for the mediating influence of “educational expectations–educational anxiety–parental involvement–child development” is (0.015, 0.055). Evidently, the confidence interval excludes the value of 0. This finding also confirms the idea of Hypothesis 4. Path analysis suggests that educational expectations of parents enhance both parental educational anxiety and parental involvement. In addition, enhancing these three factors can promote child development.

## 4. Discussion

### 4.1. Parental Educational Expectations and Child Development Are at a High Level

The results of this study, based on descriptive statistics of educational expectations and child development, revealed that the average score of parental expectations for education of young children was 4.59, with a standard deviation of 0.42. This finding signifies that parents have high educational expectations for young children, as indicated from the perspective of the mean scores. However, there still remains a certain degree of internal variability, which is consistent with the findings of prior research [57]. Some parents hold high levels of educational expectations, while some parents have only moderate educational expectations for their children. Furthermore, the mean score for child development was 4.14, with a standard deviation of 0.65. This signifies that child development was at a high level, based on the average score. However, there were still significant internal differences, with some children having a high level of development and some having an average level of development. Prior research has also discovered that children’s development in the areas of social and psychological qualities is at a high level and there is some internal variation, which aligns with the findings of this investigation [58,59].

The present study, according to the outcomes of the variance test, revealed significant differences in parental educational expectations across different class levels among children. This is consistent with previous research [57]. Specifically, the junior class exhibited the highest level of parental educational expectations, followed by the middle class. Surprisingly, the top class exhibited a comparatively modest degree of parental expectations for education. Children in the junior classes are just beginning their journey in kindergarten, and all aspects of learning and development are in their initial stages. During this time, parents hold high expectations for their children’ performance and development in kindergarten. As the level of class gradually increases and the development of the children stabilizes, parents gain a better understanding of their children’s actual performance, leading to a gradual reduction in their educational expectations.

In terms of child development, significant differences exist based on gender and region. Specifically, the level of development of girls is slightly higher than that of boys. This is due to the fact that children of different genders show different development situations and patterns at a specific stage. At the kindergarten level, girls show a higher level of development. Regionally, there are variations in children’s development levels, with the order being Xinjiang > Chongqing > Liaoning > Sichuan. Different regions have different political, economic, and cultural development conditions. Moreover, children in each region have different access to educational resources and environments, which leads to differences in their development. It is worth noting that the differences between genders and regions found in this study are different from some previous studies [60]. These differences may be due to the source of the study sample and the cultural background.

### 4.2. Parental Educational Expectations Positively Predict Child Development

The present study examines the link between the expectations for education of parents and child development in the context of society’s “educational involution”. In addition, the study seeks to investigate the fundamental mechanisms that lead to this correlation. The findings suggest that the percentage of direct effects of educational expectations on children’s development is 38%, which is very strong. Parental educational expectations have a strong positive effect on child development, which is aligned with prior research [61].

Drawing from the well-established expectancy effect (also known as the Rosenthal effect), teachers’ expectations or predictions can influence students’ performance. When teachers hold positive expectations for their students, their achievement and behavior tend to improve [7]. Similarly, this effect also applies to parents and children. As one of the most important influences in the home-education environment, parental educational expectations affect children’s development to a large degree. Although expectations for education of parents have their own uniquely self-evident effects, parents who hold high educational expectations for their children often witness positive outcomes [62]. Their children tend to develop well and show higher academic achievement in order to achieve higher levels of educational achievement compared to parents with lower expectations [63].

In this study, parental expectations for education were higher, and as a result, the children developed better. This phenomenon may be attributed to the prevailing context of “educational involution” today [2]. The mentality of young children’s parents of “hoping that their sons will grow up to be a dragon and their daughters will become a phoenix” (a Chinese saying, meaning that all parents expect their children to be successful) has become more and more serious [3]. These high expectations, coupled with the mentality of comparison among parents, contribute to the overall elevation of educational expectations. Such high educational expectations have prompted parents to adopt various methods to promote children’s development. The present study demonstrates that within the context of society’s “educational involution”, the high level of parental expectations for education significantly promotes children’s development. Consequently, this study recommends that children’s development be promoted by increasing the level of parental expectations for education.

### 4.3. Educational Anxiety Mediates the Relationship between Parental Educational Expectations and Child Development

Many studies have concluded that there is a link between educational anxiety, educational expectations and child development. In their research, scholars such as Luo found that parental expectations for education had a strong positive predictive influence on educational anxiety. These expectations were a direct cause of educational anxiety formation [64]. Research has also demonstrated that parental educational-expectation deviation significantly affects educational anxiety, and educational-expectation deviation can exacerbate educational anxiety [65]. Additionally, studies have signified that parental educational anxiety affects the development of children. On the one hand, parental educational anxiety directly and positively impacts children’s academic burnout. Specifically, as the level of parental educational anxiety increases, the severity of children’s academic burnout also increases, which hinders the children’s academic progress [66,67]. On the other hand, moderate anxiety in the family environment, with good parenting style and high educational involvement, can achieve better results, which can promote the development of children [38].

Unlike previous studies, this study further explored the mechanism of educational anxiety between educational expectations and child development. Specifically, we verified the mediation role of educational anxiety between educational expectations and child development. In the mediation analysis, the coefficient of the educational anxiety variable is 0.041, representing a contribution of 6.7% to the overall effect. The effect is mild, but this mediating effect will still play a role in this mediation model. In an era of educational involution and intense competition, many parents begin to plan for their children’s future when they are in kindergarten [6]. These parents have elevated educational expectations for their children. However, such high educational expectations often lead to parental educational anxiety, which prompts parents to adopt various behaviors to foster children’s development [6,38]. The significance of understanding the mediation role of educational anxiety lies in recognizing that parental educational expectations impact child development through subjective feelings and emotional responses, such as educational anxiety.

### 4.4. Parental Involvement Mediates the Relationship between Parental Educational Expectations and Child Development

The findings of this investigation indicate that parental involvement has a highly significant mediating effect on the link between educational expectations and child development. In the mediation analysis, the coefficient of the parental involvement variable is 0.314, representing a contribution of 50.2% to the overall effect. The effect is very strong, and plays an important role in this mediation model. Compared to a previous study, this effect is higher than the 37% reported by a previous scholar in her study [22]. This finding signifies that parents’ educational expectations influence children’s development through parental involvement. 

There exists a strong positive association between parental expectations for education and parental involvement. The degree of parents’ educational expectations determines parents’ involvement in their children’s upbringing, serving as an important factor influencing families’ parenting inputs [41]. When parents hold high educational expectations, they reinforce their own educational behaviors, adopt positive parenting input, and give more investment and attention to their children’s education. Conversely, parents with a lower level of educational expectations are generally less engaged in their children’s educational activities. They pay less attention to their children, thus hindering their children’s development. Parental involvement can positively largely influence children’s development [43]. Many studies have indicated that parents contribute financial input to promote children’s development in the hope of creating more and better educational opportunities for their children, such as enrolling their children in out-of-school educational services [68]. There are also some parents who influence children’s learning behaviors and attitudes through non-material educational support and involvement behaviors, thus promoting children’s growth and development.

Therefore, this study argues that parental involvement acts as the mediator between educational expectations and child development. For parents’ high educational expectations to be effective in promoting child development, diversified parental involvement should be increased [50]. At the material level, parents can increase their parenting investment by buying books for their children and letting their children participate in interest classes [49]. At the non-material level, parents can participate more in parent–child activities in kindergarten, and strengthen communication and exchange with their children, so as to increase their support and attention to their young children.

### 4.5. Chain Mediation Role of Educational Anxiety and Parental Involvement

This study has uncovered a correlation between educational anxiety and parental involvement, which in turn had an impact on parental educational expectations and child development. The coefficient and mediation-effect value for this relationship were 0.032 and 5.1%. The effect is mild, but this mediating effect will still play a role in this mediation model. It has been shown that as parental educational anxiety increases, families will increase the cost of child rearing and alleviate parental anxiety by investing more in upbringing [50,51]. Against the background of the “educational involution” in society, and under the influence of Chinese traditional concepts such as “expecting one’s son to be a dragon” (a Chinese saying, which means that all parents expect their children to be successful) and “a good scholar can become an official” (a Chinese saying, which means that people who study well can find a good job), many parents hold high expectations for education for their children [2,3]. They hope that they will be able to achieve better growth and development, and gain a foothold in society in the future. However, these high educational expectations often lead to serious educational anxiety among parents. To alleviate their educational anxiety, parents increasingly invest in both material and spiritual aspects of their children’s upbringing [51]. On the one hand, the increased investment in upbringing has largely eased parents’ educational anxiety. On the other hand, it has successfully facilitated the growth and development of children, while fulfilling the educational objectives of parents.

This research indicates that young children with higher parental educational expectations are more likely to achieve better development. Consequently, in our competitive environment of society, parents should raise their educational expectations for their children. In the face of educational anxiety caused by high educational expectations, parents need to increase parenting input in various aspects. By providing more attention, support, and active participation in their children’s lives, parents can foster their children’s growth and development. This increasing parental involvement serves as an effective means to alleviate educational anxiety [8,9,10]. This approach not only relieves parents’ apprehension regarding their children’s education, but also promotes the children’s development and fulfills parents’ expectations for education.

Overall, educational expectations, educational anxiety and parental involvement have a significant impact on child development. In addition, parental expectations for education can indirectly influence child development through their effects on educational anxiety and parental involvement. Moreover, apart from these factors, there are other aspects that may affect child development, including region, gender, and family socioeconomic status (SES). These variables still deserve our attention, so as to better promote child development.

### 4.6. Research Limitations and Prospects

The results of this study have the capacity to offer significant direction to the government, society, kindergartens, and education service organizations. Specifically, these insights can assist parents in promoting child development. For example, kindergartens can provide more opportunities for parents to participate in their children’s daily activities. By doing so, they enhance parental involvement, which contributes to positive child development.

However, this study is subject to many constraints. First, this study employed a cross-sectional design and did not use a longitudinal study to investigate the correlation between parental educational expectations, educational anxiety, parental involvement, and child development. Second, this study employed a questionnaire to gather information from parents, a method that could potentially be influenced by subjective factors. Third, the sample used in this study was from four provinces, which is not representative enough. It needs to be further examined to see if it can be generalized to more regions.

Future studies can explore longitudinal research by expanding the scope and number of sample areas, adopting a more objective survey approach, and incorporating other relevant variables.

## 5. Conclusions

In conclusion, although previous research has provided possible directions for the link between parental expectations for education and child development, the underlying mechanisms remain elusive. Expanding and deepening previous research, the current investigation analyzed the impacts of parental educational expectations on child development in the context of the current social context of “educational involution”. The findings suggest that educational expectations have a significant predictive impact on child development. Educational expectations can indirectly influence child development through the mediation effects of educational anxiety and parental involvement. These empirical findings can function as a reliable reference for governments, kindergartens, and educators, to support parents to promote child development. For parents of young children, they will also be able to find effective ways to promote their children’s development.

## Figures and Tables

**Table 1:** The demographic characteristics of participants.

Variables	Options	N (%)	M ± SD
Gender of the child	Boy	403 (52.5)	-
	Girl	364 (47.5)	-
Only child or not	Yes	307 (40.0)	-
	No	460 (60.0)	-
Region	Sichuan Province	276 (36.0)	-
	Chongqing Municipality	164 (21.4)	-
	Liaoning Province	156 (20.3)	-
	Xinjiang Uygur Autonomous Region	171 (22.3)	-
Child’s class level	The junior class	320 (41.7)	-
	The middle class	249 (32.5)	-
	The top class	198 (25.8)	-
Location of the child’s household	Cities and towns	289 (37.7)	-
	Countryside	478 (62.3)	-
SES	-	-	0.00 ± 0.78

Note: SES = family socioeconomic status; M ± SD = mean ± standard deviation.

**Table 2:** The descriptive statistics and correlation analysis of main variables.

Variables	M ± SD	1	2	3	4	5
1. EE	4.59 ± 0.42	1	-	-	-	-
2. EA	3.80 ± 0.89	0.196 ***	1	-	-	-
3. PI	4.10 ± 0.61	0.407 ***	0.281 ***	1	-	-
4. CD	4.14 ± 0.65	0.386 ***	0.311 ***	0.635 ***	1	-
5. SES	0.00 ± 0.78	0.071 *	−0.174 ***	−0.080 *	−0.074 *	1

Note: *** *p* < 0.001, * *p* < 0.05; M ± SD = mean ± standard deviation; EE = educational expectations; EA = educational anxiety; PI = parental involvement; CD = child development; SES = family socioeconomic status.

**Table 3:** The one-way ANOVA and independent samples *t*-tests.

Variables		Educational Expectations	Child Development
Gender of the child	Boy (M ± SD)	4.59 ± 0.45	4.09 ± 0.67
	Girl (M ± SD)	4.60 ± 0.38	4.20 ± 0.62
	F	1.117	1.909
	*p*	0.728	0.017 *
Only child or not	Yes (M ± SD)	4.59 ± 0.39	4.09 ± 0.62
	No (M ± SD)	4.59 ± 0.43	4.17 ± 0.67
	F	0.370	1.641
	*p*	0.841	0.088
Region	Sichuan Province (M ± SD)	4.60 ± 0.47	3.91 ± 0.70
	Chongqing Municipality (M ± SD)	4.61 ± 0.25	4.29 ± 0.53
	Liaoning Province (M ± SD)	4.53 ± 0.51	4.08 ± 0.66
	Xinjiang Uygur Autonomous Region (M ± SD)	4.60 ± 0.34	4.40 ± 0.50
	F	1.171	25.828
	*p*	0.320	<0.001 ***
	LSD	-	SC < LN < CQ < XJ
Child’s class level	The junior class (M ± SD)	4.64 ± 0.37	4.10 ± 0.66
	The middle class (M ± SD)	4.58 ± 0.41	4.12 ± 0.64
	The top class (M ± SD)	4.54 ± 0.49	4.22 ± 0.64
	F	3.724	2.003
	*p*	0.025 *	0.136
	LSD	T < M < J	-
Location of the child’s household	Cities and towns (M ± SD)	4.58 ± 0.43	4.16 ± 0.66
	Countryside (M ± SD)	4.60 ± 0.41	4.12 ± 0.64
	F	3.127	1.492
	*p*	0.434	0.447

Note: *** *p* < 0.001, * *p* < 0.05; M ± SD = mean ± standard deviation; LSD = least significant difference; SC = Sichuan Province; CQ = Chongqing Municipality; LN = Liaoning Province; XJ = Xinjiang Uygur Autonomous Region; J = the junior class; M = the middle class; T = the top class.

**Table 4:** The stratified regression results.

Variables	E	STE	E	STE	E	STE	E	STE
Constant	1.378	-	1.335	-	1.123	-	1.045	-
Educational Expectations	0.601	0.386	0.600	0.385	0.598	0.384	0.613	0.393
Gender ^a^	-	-	0.105	0.081	0.087	0.067	0.087	0.067
Region ^a^	-	-	-	-	0.375	0.238	0.415	0.263
Region ^b^	-	-	-	-	0.206	0.128	0.198	0.123
Region ^c^	-	-	-	-	0.476	0.306	0.496	0.319
SES	-	-	-	-	-	-	−0.121	−0.146
R^2^	0.149		0.156		0.244		0.265	
F	133.941 ***		70.390 ***		49.149 ***		45.572 ***	
ΔR^2^	0.149		0.148		0.147		0.153	
ΔF	133.941		134.078		147.669		158.415	

Note: *** *p* < 0.001; SES = family socioeconomic status; E = coefficient; STE = standardized coefficient; Gender ^a^ = dummy variable, refers to the comparison of girls and boys; Region ^a^ = dummy variable, refers to the comparison of Chongqing Municipality and Sichuan Province; Region ^b^ = dummy variable, refers to the comparison of Liaoning Province and Sichuan Province; Region ^c^ = dummy variable, refers to the comparison of Xinjiang Uygur Autonomous Region and Sichuan Province.

**Table 5:** The path analysis.

Paths	SC	SE	*p* Value
Educational Expectations → Educational Anxiety	0.210	0.074	<0.001
Educational Expectations → Parental Involvement	0.417	0.046	<0.001
Educational Expectations → Child Development	0.393	0.049	<0.001
Educational Anxiety → Parental Involvement	0.245	0.024	<0.001
Educational Anxiety → Child Development	0.265	0.025	<0.001
Parental Involvement → Child Development	0.592	0.030	<0.001

Note: SC = standardized coefficient; SE = standard error.

**Table 6:** Mediation effects of main variables.

Models	Paths	Effect	Boot SE	Boot LLCI	Boot ULCI	
Direct Effect	EE → CD	0.237	0.047	0.146	0.329	38%
Indirect Effect	EE → EA → CD	0.041	0.015	0.017	0.074	6.7%
	EE → PI → CD	0.314	0.038	0.240	0.387	50.2%
	EE→ EA → PI → CD	0.032	0.010	0.015	0.055	5.1%
Total Effect	-	0.625	0.051	0.525	0.724	100%

Note: SE = standard error; CI = confidence interval; EE = educational expectations; EA = educational anxiety; PI = parental involvement; CD = child development.

## Data Availability

The raw data supporting the conclusions of this article will be made available by the authors on request.
